# Early Evidence of Acheulean Settlement in Northwestern Europe - La Noira Site, a 700 000 Year-Old Occupation in the Center of France

**DOI:** 10.1371/journal.pone.0075529

**Published:** 2013-11-20

**Authors:** Marie-Hélène Moncel, Jackie Despriée, Pierre Voinchet, Hélène Tissoux, Davinia Moreno, Jean-Jacques Bahain, Gilles Courcimault, Christophe Falguères

**Affiliations:** 1 Department of Prehistory-UMR 7194 CNRS, National Museum of Natural History, Institut de Paléontologie Humaine, Paris, France; 2 Bureau de Recherches Géologiques et Minières, GEO/G2R, BP 36009, Orléans, France; Illinois State University, United States of America

## Abstract

The human settlement of Europe during Pleistocene times was sporadic and several stages have been recognized, both from paleaoanthropological and archaeological records. If the first phase of hominin occupation (as early as 1.4 Ma) seems mainly restricted to the southern part of the continent, the second phase, characterized by specific lithic tools (handaxes), is linked to Acheulean settlements and to the emergence of *Homo heidelbergensis*, the ancestor of Neanderthals. This phase reached northwestern Europe and is documented in numerous sites in Germany, Great Britain and northern France, generally after 600 ka.

At la Noira (Brinay, Central France), the Middle Pleistocene alluvial formation of the Cher River covers an archaeological level associated with a slope deposit (diamicton). The lithic assemblage from this level includes Large Cutting Tools (LCTs), flakes and cores, associated with numerous millstone slabs. The lithic series is classified as Acheulean on the basis of both technological and typological analyses. Cryoturbation features indicate that the slope deposits and associated archaeological level were strongly frozen and disturbed after hominin occupation and before fluvial deposition. Eight sediment samples were dated by the electron spin resonance (ESR) method and the weighted average age obtained for the fluvial sands overlying the slope deposits is 665±55 ka. This age is older than previous chronological data placing the first European Acheulean assemblages north of 45^th^ parallel north at around 500 ka and modifies our current vision of the initial peopling of northern Europe. Acheulean settlements are older than previously assumed and the oldest evidences are not only located in southern Europe. La Noira is the oldest evidence of Acheulean presence in north-western Europe and attests to the possibility of pioneering phases of Acheulean settlement which would have taken place on a Mode 1-type substratum as early as 700 ka. The lithic assemblage from la Noira thus provides behavioral and technological data on early Acheulean occupation in Europe and contributes to our understanding of the diffusion of this tradition.

## Introduction

Discoveries made over the past two decades attest to a large diversity of Mode 1-type assemblages as early as 1.4 Ma ago in Europe (e.g. Orce, Atapuerca, Pirro-Nord [1], [2]). The scarcity of sites over so long a period of time suggests short-lived dispersal events and probably a source-sink dynamic from the south with phases of depopulation and recolonization. Assemblages without evidence of bifacial technology persist after 1 Ma and new discoveries in the center of France and in England enhance our vision of human colonization in northern parts of the continent [3], [4], [5], [6], [7]. The first assemblages with Large Cutting Tools (LCTs) (Mode 2, cf. Clark [8]) are described in East Africa from 1.8 Ma and in the Levant and India from 1.5 Ma [9], [10], [11], [12], [13], [14], and relationships between African, Levantine and Indian sites are acknowledged [15]. In China, LCTs at 800 ka suggest a local or allochthonous origin [16], [17].

The first assemblages with bifacial tools in Europe are much younger. The usual hypothesis is that the first Acheulean evidence appears at around 600 ka in southern Europe, followed by a rapid colonization of the northwest at 500 ka [18], [19], [20], [21]. This first occurrence could be older [22], but this has not yet been firmly established (sites of Solana del Zamborino and Cueva Negra Quipar). The Acheulean tradition would thus have been the first to continuously occupy northern latitudes.

The few hominin fossils dating between 800 and 500 ka (Gran Dolina TD6, Mauer, Boxgrove) are attributed to either *Homo antecessor* or *Homo heidelbergensis* and diversity and ambiguities of anatomical features are noted [23], [24], [25], [26], [27], [28], [29], [30], [31]. Dental analysis points to longitudinal migrations of new hominin groups from Asia, and genetic data suggest speciation events in Africa or Eurasia prior to 600 ka [32], [33], [34].

The 800 to 500 ka timescale is a key period in Europe since series with (Notarchirico and Arago levels P-Q) and without bifacial tools (Pakefield, Happisburg I, Soleilhac, Isernia la Pineta, Atapuerca Gran Dolina TD6) co-occurred. New discoveries at la Noira site, located beyond the 45^th^ parallel north in the center of France, enhance our vision of both the northern colonization of Western Europe and the earliest bifacial traditions [35], [36], [37]. Evidence from la Noira raises the question of the relationship between *Homo heidelbergensis* dispersals and the onset of the Acheulean and LCTs.

The study of the lithic assemblage of la Noira using technological and typological analytic methods (e.g. [38], [39], [40], [41], [42] to define the techno-economic processes, manual dexterity, technological sequences (*chaîne opératoire*) and technological skills involved in tool production contributes to our knowledge of hominin group cognition in Europe during the period when *Homo heidelbergensis* appeared.

## Materials and Methods

### 1.The Middle Cher River Valley

**Figure 1:**
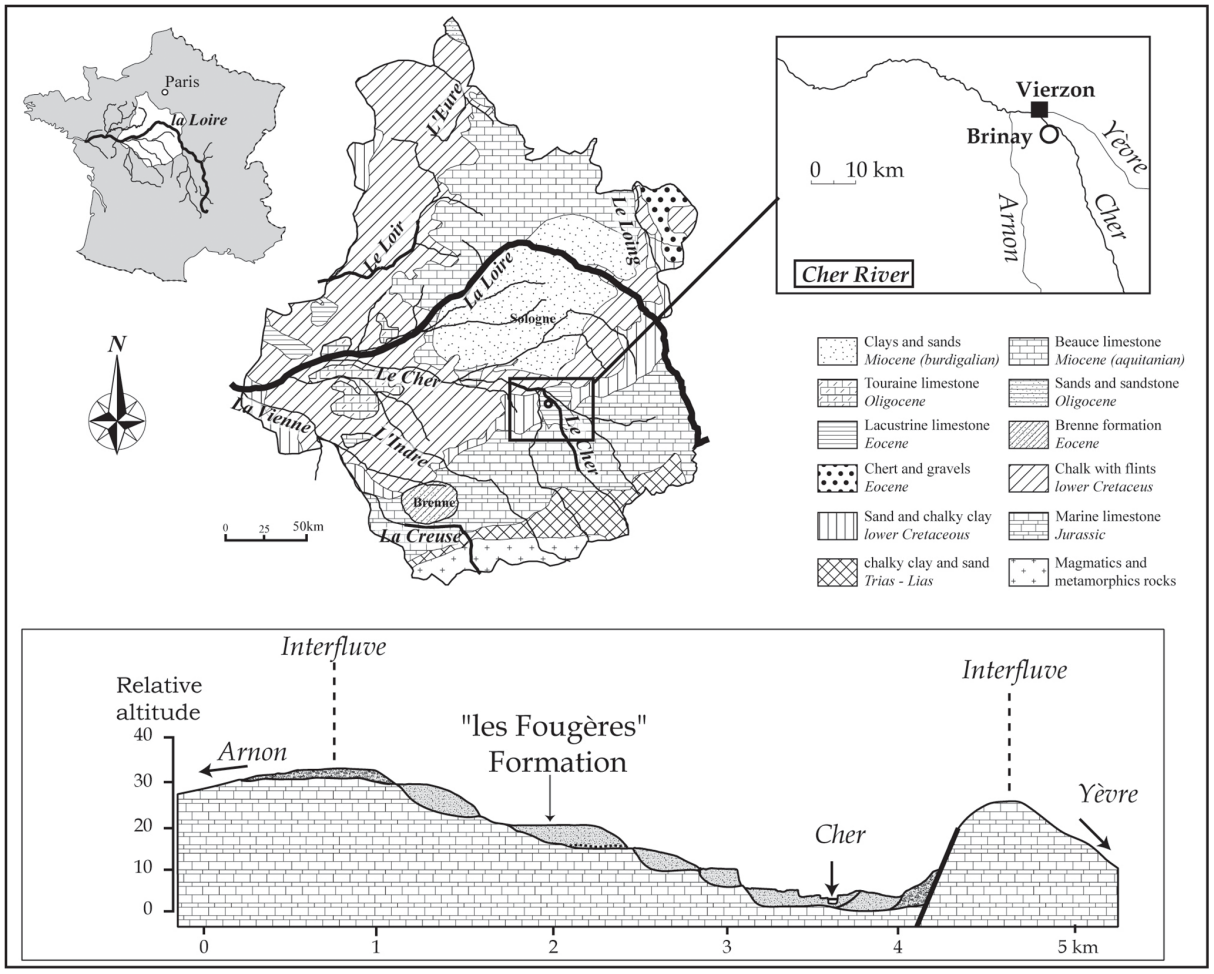
Geographic and geological maps showing the location of the la Noira Site at Brinay, (Cher, France) in the Middle Cher River valley and the location of the Les Fougères fluvial formation on the western slope of the valley. The figure is for illustrative purposes only.

**Figure 2:**
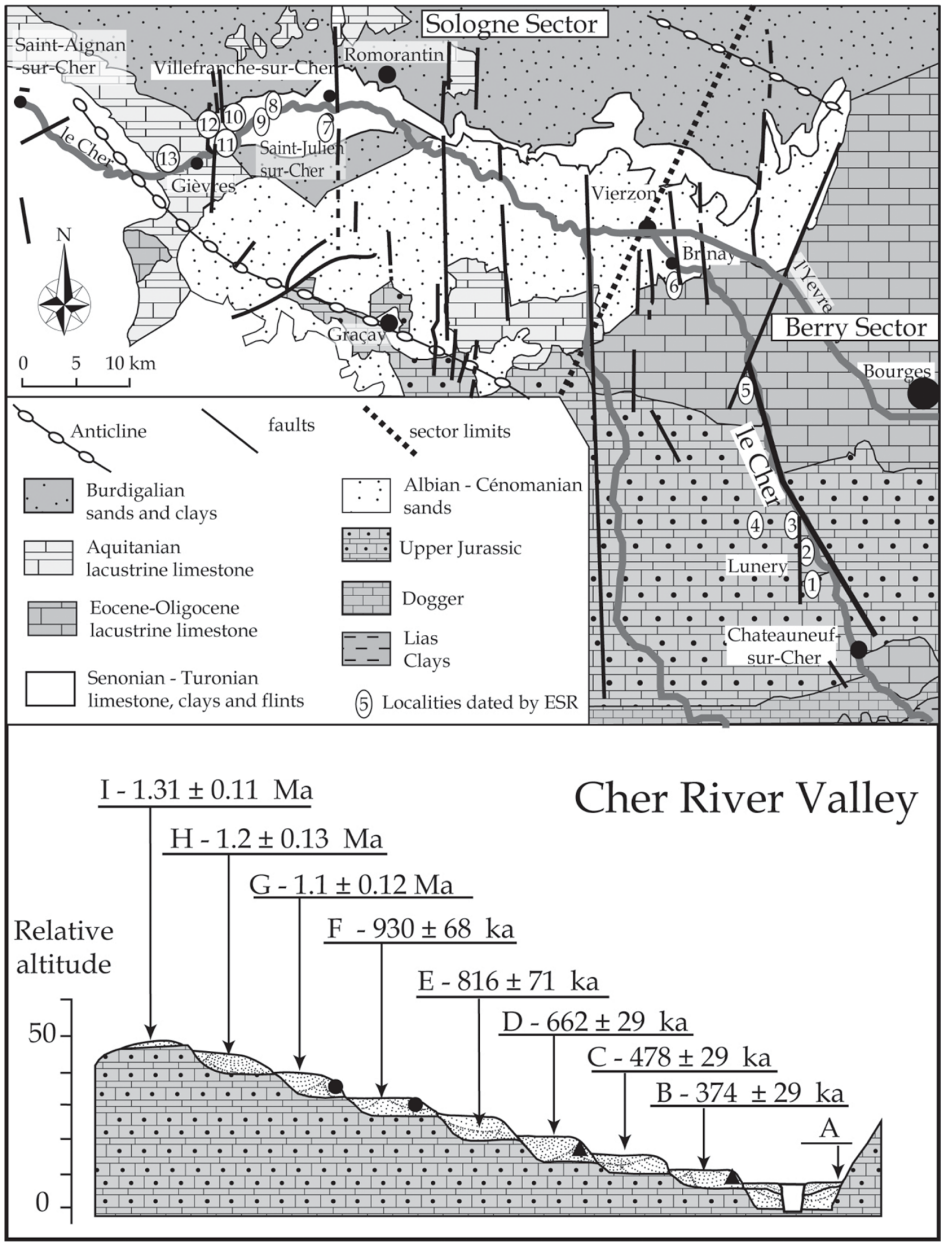
Geological map of the Vierzon area and situation of the Middle Cher Valley and theoretical transect of the Middle Cher valley, and ESR ages of the eight fossil fluvial formations. (rounds : Early Palaeolithic sites, triangles : Lower Palaeolithic sites). The figure is for illustrative purposes only.

The site of la Noira is located in the Middle Cher River Valley, a tributary of the Loire River in the Middle Loire Basin (Centre region, France) near Brinay village (Figures 1, 2). In this area, the fossil fluvial system of the Cher River is composed of nine stepped alluvial formations [37] deposited during successive fluvial incisions and aggradations as a response to climatic cyclicity during the Early and Middle Pleistocene [35]. This stepped disposition is also related to a slow uplift of the Paris Basin, as described by Antoine et al. [43] for river systems in the northern Paris Basin.

### 2.The site of la Noira

The site of la Noira was discovered at the beginning of the 1970s in a quarry. Since 2003, systematic geological and geochronological studies have been applied to the Pleistocene depositional sequence, supported by the French Ministry of Culture and an ANR project. Field work permission for la Noira site was granted to Jackie Despriée and Marie-Hélène Moncel by the French Ministry of Culture. The quarry owner granted permission to conduct fieldwork and this study. The site is located in the Les Fougères Formation, which is one of the stepped Pleistocene fluvial sheets deposited by the Cher River (sheet D) lying on the western slope (left bank) of the valley. It is a thick fluvial sandy formation, about 1.250 km long and 0.5 km wide. This terrace was deposited on an Oligocene lacustrine limestone block delimited by two west-east ground features. The fluvial formation is deposited between a relative altitude of +13 m and +21 m above the last level of incision under the present alluvial plain.

**Figure 3:**
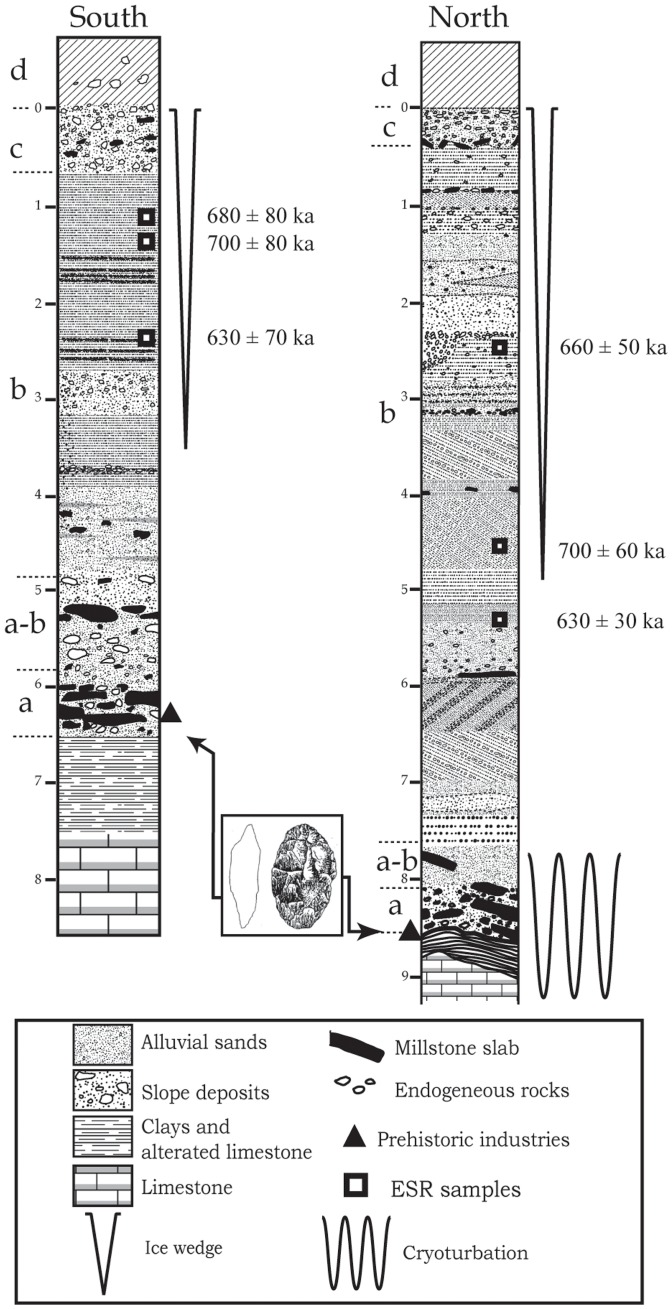
Positions of ESR samples (black squares) taken in 2003 (South Log) and in 2006 (Northern Log) and ages obtained. The black triangles indicate the location of the prehistoric levels observed in situ during quarrying, sampling or excavating. The figure is for illustrative purposes only.

Above the substratum of Stampian lacustrine limestone weathered in green clay, the Les Fougeres Quaternary formation bears five successive strata: a coarse slope deposit or diamicton (stratum a) covered by two sequences of sandy alluvial layers (stratum b), then a rubble level (stratum c) and a silty soil (stratum d). The archaeological level studied in this article is located in stratum a (Figure 3).

**Figure 4:**
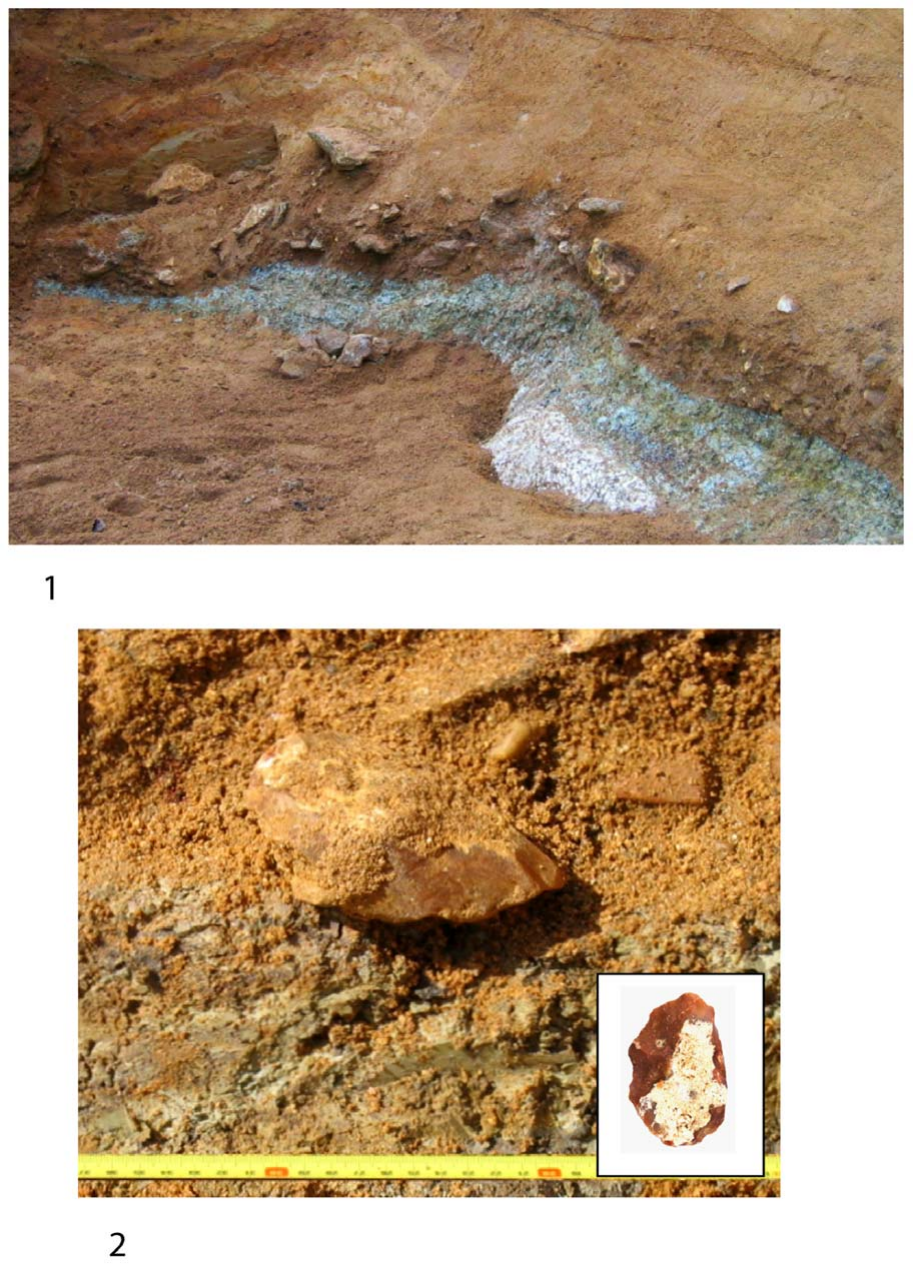
Photos of the stratum a with a biface *in situ*. 1.View of stratum a, with cryoturbated masses of millstone slabs and artefacts on weathered limestone. 2. View of a biface *in situ* at the bottom of a cryoturbated feature (cf. Despriee et al., 2007).

The sedimentation history seems similar to that described by Antoine et al for the Somme valley [43]. In the la Noira quarry, the observed sedimentary formation was approximately six to eight meters thick (see Figure 3). Firstly, the basal layer (stratum a) was deposited during an early glacial stage after the down cutting of the valley. It is a coarse slope deposit which contains masses of local millstone slabs deposited on the clayey bedrock (Tertiary limestone), mixed with magmatic, metamorphic, crystalline and sedimentary rocks, and covered with coarse quartz sand in a brown rubefied clayey matrix with numerous iron pisoliths and weathered blocks or pebbles of endogenous rocks. Some of the siliceous slabs, which range in size from several centimeters to several decimeters, bear scars attesting hominin activities [6] (Figure 4). These materials (granite, gneiss, quartz, sandstones, lacustrine millstones…) were spread and accumulated during Tertiary epochs (Oligocene to Pliocene) by torrents or wadis on the peneplain areas at the southern edge of the Massif Central. These detrital formations were lateritized during Oligocene stages. During the Quaternary period, after each down cutting of the Cher River, at the beginning of a cold climatic stage, these coarse sediments came down slope by solifluction from the interfluve or the edges of the plateau, and were stocked on the floor of the new alluvial plain. The artifacts are associated with abundant millstone slabs, generally lying flat on the clayey floor.

After hominin activities, part of stratum a - enclosing the archaeological pieces - and the underlying limestone bedrock were cryoturbated before the sandy alluvial deposition. Despite this reworking, an undisturbed archaeological area from stratum a, containing many artefacts, is currently undergoing excavation. After this human occupation, the Cher River deposited gravels and sands as new slope coarse deposits (gelifuction) above the diamicton, during the same glacial stage and the next interglacial period, before the following incision of the valley.

About 300 Electronic Spin Resonance (ESR) samples were taken in the fossil sandy alluvial deposits of the three middle valleys of the Loir, Cher and Creuse Rivers in the Loire catchment basin. Dating was carried out at the National Museum of Natural History, Paris, France.

## Results

### 1.Dating

**Figure 5:**
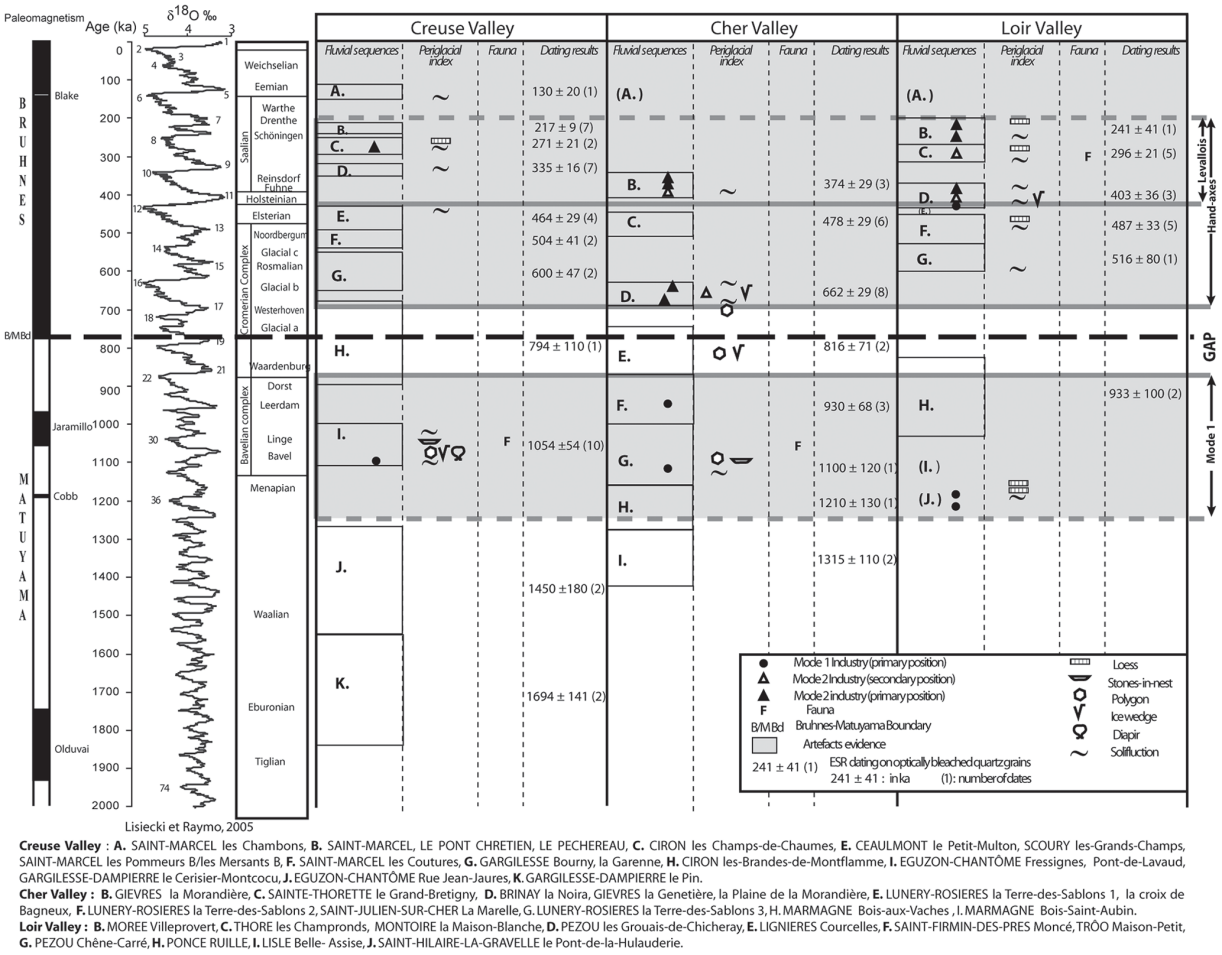
Chronological framework for the river systems of the Creuse, Cher and Loir valleys, ESR ages of river aggradation, location of prehistoric artefacts in primary or secondary position, and evidence of periglacial phenomena. La Noira Acheulean workshops are situated in MIS 16, as shown by evidence of periglacial phenomena, before covering by interglacial fluvial deposits during MIS 15.

The results enable us to place the stepped formations of each fluvial system (and the prehistoric sites covered by these formations) within Early and Middle Pleistocene chronological frameworks based on oxygen isotope variations (Figure 5). In the Cher Valley, ESR applied to optically bleached quartz was used to date more than 80 samples [35], [6], [37].

At la Noira, the very acidic quartzose deposits are not conducive to the preservation of organic remains, and the age of the site was therefore determined using the ESR method applied to optically bleached sedimentary quartz grains. Since 2003, ESR samples have been taken from stratum b at la Noira site. The ESR age obtained for stratum b, corresponding to a fluvial deposit, provided a mean age value of 655±55 ka, [35], [37] (Table 1). The la Noira site is the only currently known prehistoric site discovered during the time period ranging between 700 and 600 ka in the Centre Region [6].

**Table 1:** ESR dates from the la Noira sequence.

Samples	Stratum	Depth	ESR ages
Cher 2004-12	Stratum b (top)	1.10 m–1.20 m	680±80 ka
Cher 2004-11	Stratum b (top)	1.20 m–1.45 m	700±80 ka
Cher 2004-10	Stratum b (top)	2,40 m–2.50 m	630±70 ka
Cher 2006-2	Stratum b (median)	2.50 m–2.60 m	660±50 ka
Cher 2006-1	Stratum b (median)	4.50 m–4.60 m	700±60 ka
Cher 2006-3	Stratum b (bottom)	5.20 m–5.30 m	630±30 ka

The hominin occupation occurred between the end of the river incision and the fluvial deposits and suggests that hominins were present during the early glacial stage, just before the pleniglacial phase and before interglacial fluvial deposition [6]. This glacial/interglacial cycle can be assigned to the MIS 16/MIS 15 cycle, according to the ESR results obtained for the fluvial layers.

### 2.Lithic assemblage of la Noira, stratum a

The lithic assemblage is located in the coarse stratum a beneath the sandy fluvial sequence.

The stratum a level yielded a series of 340 items found *in situ* in two sectors of the site during fieldwork conducted between 2003 and 2012. Only pieces with evidence of manufacture have been analyzed and five rolled cores from an upper and older formation were discarded. The assemblage is composed of cores, flakes, crude core-tools and Large Cutting Tools (LCTs), which make up 17% of the series.

#### 2.1.Raw materials

The main raw materials used are local millstone slabs (90%) (Table 2). The millstone slabs shaped or flaked by hominins are issued from diagenetic silicifications included in the Oligocene lacustrine limestone that forms the substratum of the Vierzon area, and, at Brinay, the plateau and the slopes of the Cher valley. These silicifications form parallelepiped slabs of up to several decimeters, generally from five to ten centimeters thick.

**Table 2:** Main lithic types in stratum a at la Noira.

Artefacts	Millstone	Jurassic Oolithic chert
Crude pieces	20	-
Cores	57 (40 slabs, 2 flakes, 2 nodules)	1 (nodule)
Flakes	199	6
Large cutting tools	58 (including 2 flakes)	-

These silicified slabs are found lying in a horizontal position in the lacustrine limestone formation. The process of silicification fossilized original and various facies types deposited at the bottom of the lacustrine area in calm and shallow freshwater. Brecciated structures, algal and stromatolitic deposits, vermiculated cavities, burrows and tubing with casts of roots and plant debris are visible in the millstone and affect its thickness, but it can nonetheless be flaked or shaped. Some slabs with a very fine and homogeneous siliceous mass bear no irregularities and have very good flaking qualities.

Millstones moved downslope after being exposed during river incision in the limestone formation. They were carried over a very short distance to the slope bottom, and only rare marks shorter than one millimeter are visible on ridges. No surface crushing is observed. These slope formations thus provided raw material and were used as an outcrop. Available millstone slabs were exploited *in situ* by hominins and artefacts are associated with this deposit.

Rare Jurassic oolithic chert nodules were also used by hominins. These nodules were mixed with millstone slabs, and come from the oldest alluvial formations further up the slopes.

#### 2.2.Crude cores or bifacial tools

**Figure 6:**
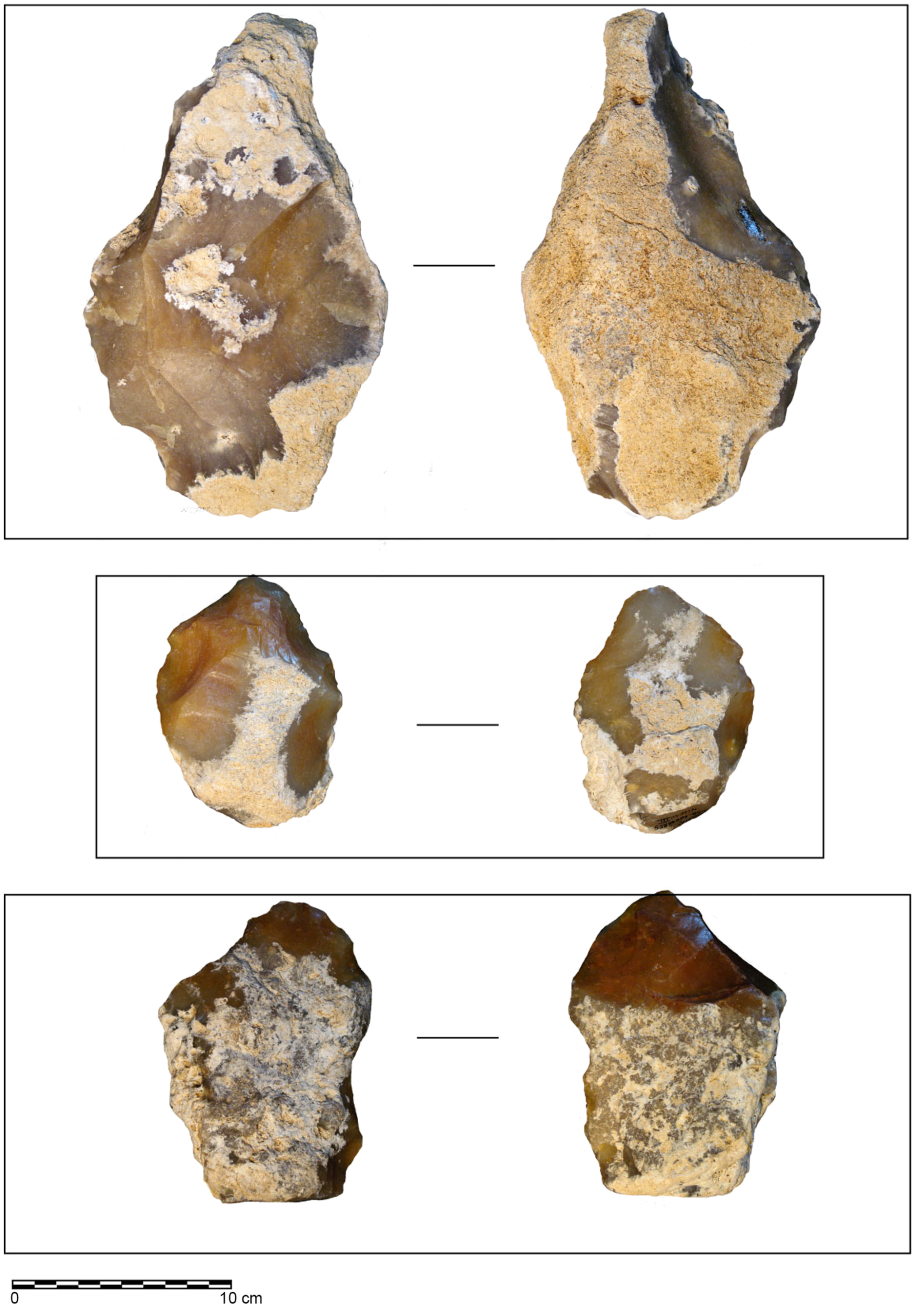
Examples of slabs with few removals. Crude cores or crude bifacial tools?

Millstone slabs (80 to 240 mm long) at various stages of manufacture and one large flake with few scars were recovered. These pieces can be attributed to cores or unfinished bifacial tools (preforms): 1) incomplete management of convergent edges and a tip indicating shaping, 2) width/thickness ratio higher than 1.5 for LCT preforms, 3) thin and invasive series of removals on LCTs, deep and short on cores. They attest to the presence of workshops with expedient *débitage* and partial shaping (Table 2, Figure 6). Hominins may have been attracted to this zone by the large quantity of available raw materials on the river bank. This site might have also recorded evidence of domestic activities, as indicated by six broken bifacial tools and the frequent partial crushing of cutting edges not due to taphonomic processes. Some LCTs were not broken during shaping; they are largely worked and retouched. We observe either a lateral percussion impact or a flexion on the fracture (from the flatter face). Moreover, a fragment of a bifacial tool was reworked as a point. The others do not display evidence of reworking.

#### 2.3.Core technology

The core technology of the assemblage indicates complex and organized techno-economic patterns and is focused on the *débitage* of medium-large flakes. Part of this *débitage* is devoted to the production of large flakes for both flaking (n = 2) and shaping (n = 2). Large flakes are distinguished from small *débitage* by their dimensions: length or width >10 cm [11], [14], [43].

The high number of cores enables us to appraise the quantitative and qualitative modalities of the knapping technology and volume management. Cores have been identified by the organization of removals. A panoply of opportunistic or structured methods using hard hammer direct percussion has been identified. Two cores bear strong crushing marks on their edges and some large quartz and metamorphic rock pebbles found *in situ* document percussion activities and have been considered to be a set of hard hammers. The cores can be divided into several groups of varied modalities with regard to the shape of the slab and the best opportunities offered. This indicates awareness of the reactions of the millstone slab properties and the relatively minor role of natural slab shape, except at the beginning of the reduction sequence.

The main method used is partial bifacial flaking on millstone slabs (44%). Each cortical surface of the slab is knapped by a single series of short deep removals using alternate or face by face flaking.

**Figure 7:**
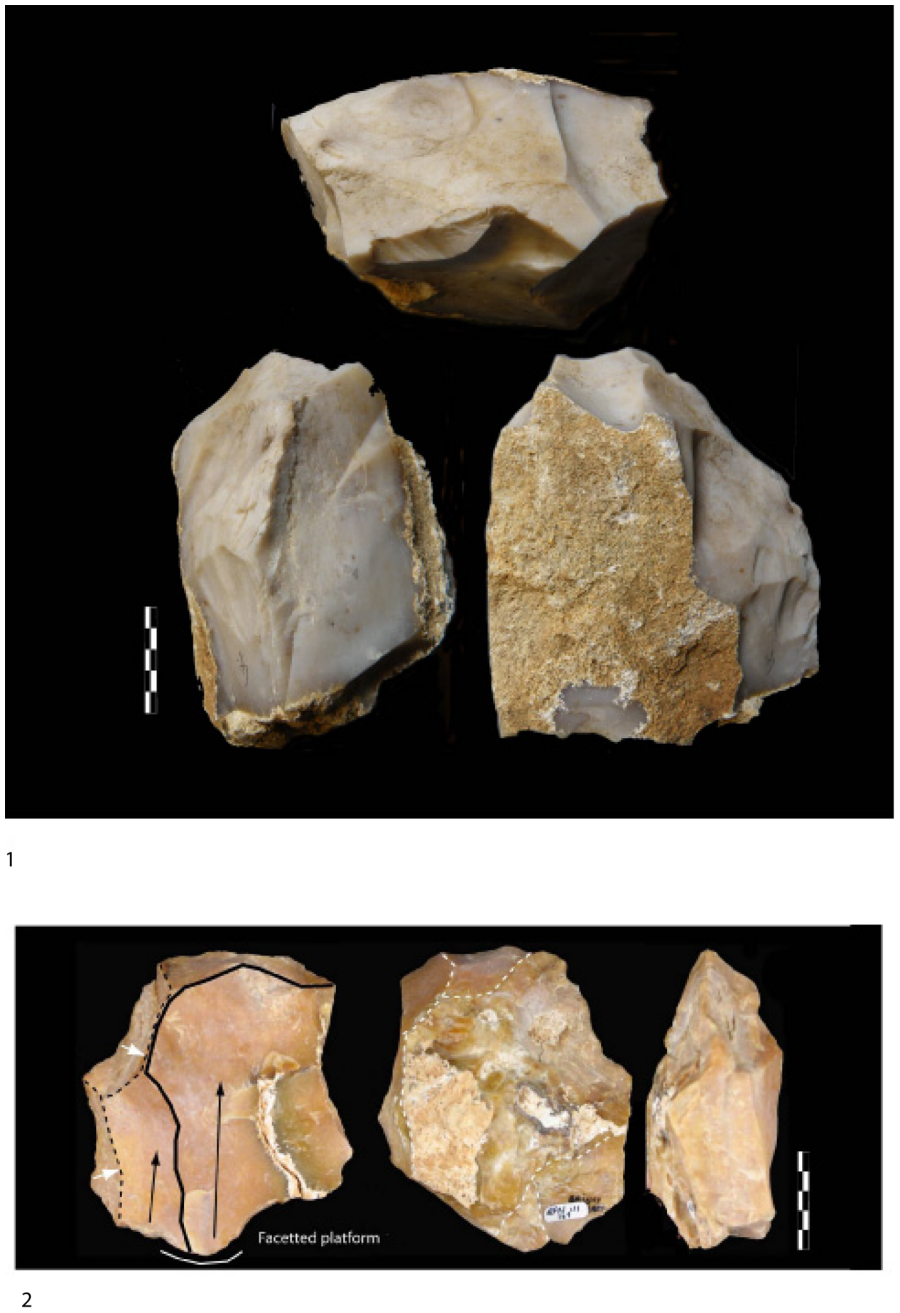
Examples of millstone cores of stratum a. 1. Partial bifacial core on millstone slab with deep removals and a partial peripheral edge. 2. Millstone bifacial core with a careful facetted configuration of the platform.

**Figure 8:**
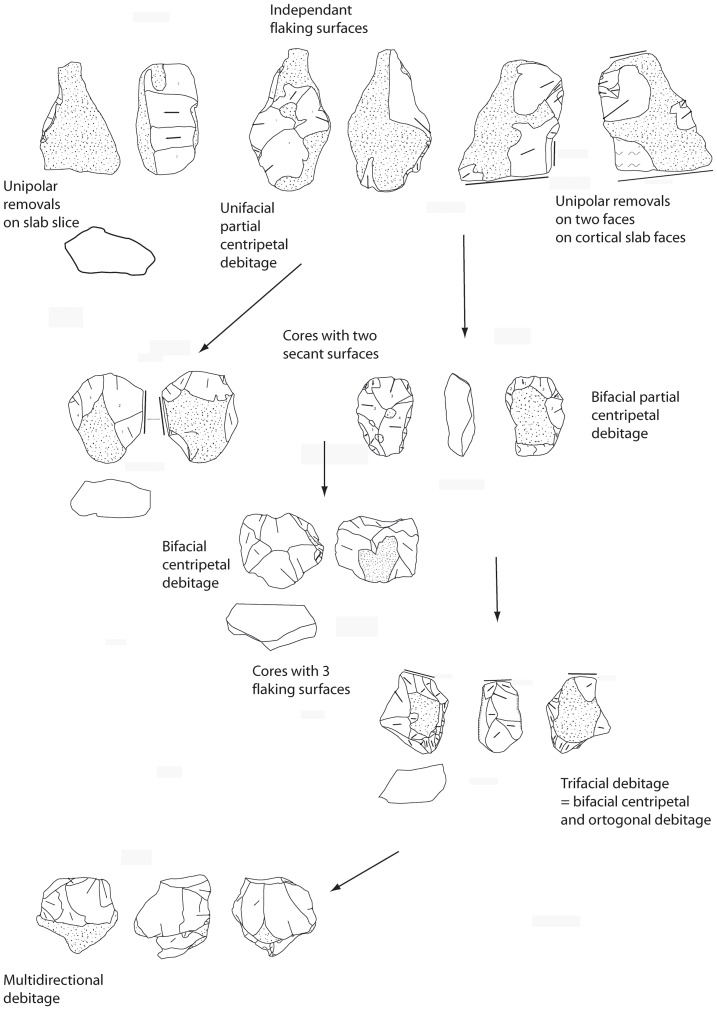
Debitage reduction sequence at stratum a at la Noira.

Two large groups of partial bifacial discoid cores can be distinguished (Table 3 and Figures 7, 8):

**Table 3:** Main core types from stratum a at la Noira.

	Millstone	Oolithic Jurassic chert	Total
Unifacial on one face or slab edge	6		6
Partial bifacial discoid cores	24	1	25
Total bifacial discoid cores	8		8
Bifacial discoid core (debitage of a surface)	1		1
Bifacial discoid or thick tool	1		1
Trifacial cores with few removals	4		4
Trifacial cores	3		3
Orthogonal simple or double	2		2
Multidirectional cores	5		5
Indeterminate cores (broken)	3		3
Total	57	1	58

1) symmetrical cores with alternating flaking resulting in a sinusoidal cutting edge (angles between 75–85°), 2) unsymmetrical cores with one flat surface due to a face by face debitage. The flat surface bears more invasive and elongated (deep or not) centripetal or crossed removals opposite another surface with short scars due to the perpendicular knapping of the slab slice (striking platform?). The secant cutting edges are more regular than for the former type. Some final removals could be produced on the abrupt surface if favorable platforms existed. Cutting edge angles are close to 70–80°. No evidence of core preparation is apparent and flaking stopped abruptly, either due to the large quantity of available slabs on the site or to the fact that the flaking surface was no longer functional with suitable angles. The core-flakes are cortical and thick (40 mm) with abrupt removals on the upper face of the flake and some scars on the lower surface (bulb).

Three groups of total bifacial cores exist:

1. pyramidal cross-section with deep and elongated or short removals on one face (85°),
2. alternate flaking on bi-pyramidal surfaces (60–70°),
3. flat surface with thin and more or less invasive and centripetal removals opposite a pyramidal surface bearing the last removals (60°). The first and second categories can be described as bifacial flaking using the most suitable angles with each previous core scar resulting in an irregular peripheral cutting edge. For the third type, as one of the surfaces is convex with centripetal removals, a flaking hierarchy is suggested. A few pieces suggest a hierarchy of the two surfaces between the platform and the flaked surface. One isolated case displays a “*chapeau de gendarme*” platform (89°) and a flat flaked surface (pre-Levallois concept *e.g.* Tyron et al. [45]. Two abrupt removals were produced beforehand on the lateral part of the core before a final invasive removal. These two lateral removals could suggest previous preparation of the lateral edge of the core. However the poor quality of the stone does not enable us to identify any kind of predetermination in core management (although this piece bears some of the technical criteria developed by the Levallois methodology). In this case, we only have evidence of hierarchical management and we are unable to distinguish stages of full debitage from possible phases of convexity preparation (Figure 7b).

Trifacial (three orthogonal flaking surfaces or a mixture of one surface and a partial bifacial flaking) and unifacial (one flaking surface) cores display expedient debitage, parallel or perpendicular to cortical slab planes. Overexploited multifacial cores are rare.

Most of the cores measure between 50 and 180 mm, indicating the coexistence of both small and large debitage. The few cores measuring close to or more than 200 mm were flaked by limited removals. The single chert core is one of the smallest pieces and was exploited in the same way as the millstone cores. Since most of the cores bear preserved cortical patches on both surfaces, we can estimate the average thickness of the flaked slabs between 40 and 90 mm, which is higher than for the LCTs. Double patina on some bifacial cores indicates recurrent occupations and the re-use of abandoned cores, including the most complex ones.

**Figure 9:**
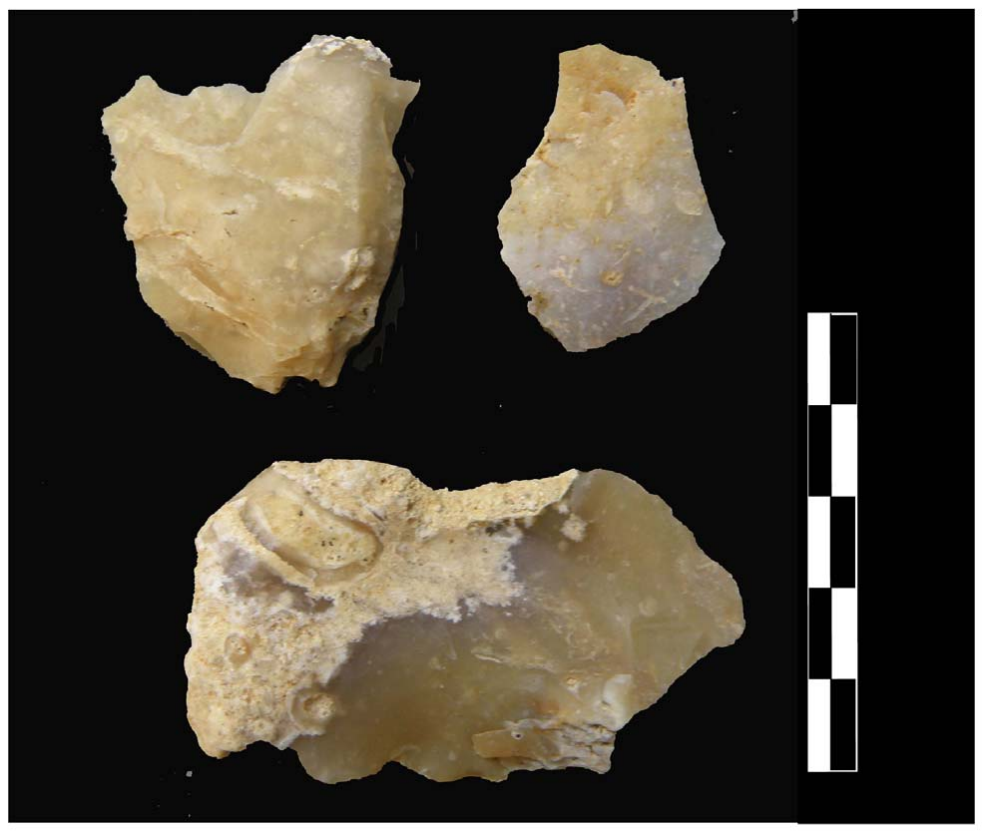
Flakes issued from shaping and flaking.

Flakes are consistent with core management and are issued from all stages of the reduction sequence (Figures 8, 9). Millstone flakes are the largest products (up to 100 and 200 mm long). Five flakes present features related to bifacial shaping using a soft hammer (evidence of lipped platform, curved profile and thin section).

21% of the flakes are retouched (n = 39), most of them being the largest millstone flakes. Thin and direct retouch does not denote the pursuit of a specific morphology. Tools are mainly single (76%), and are largely comprised of scrapers (30%), Clactonian notches and denticulates (27%).

#### 2.4.Large Cutting Tools (LCTs)

The main feature of the series is the diversity of the LCTs, most of which are made on 20–40 mm thick millstone slabs. LCTs are distinguished from retouched flakes by their dimensions [11], [45], [14]. They generally measure between 60 to 160 mm long but some attain 170–235 mm. The two flake-supports are cortical, belong to the middle-sized group and are issued from centripetal or unidirectional *débitage*. The presence of reduced (47%) or large cortical surfaces (38%) facilitates the reading of the successive removals and suggests voluntary size variations for LCTs.

Several groups of tools have been distinguished according to the type of tip management and the bifacial equilibrium between the two faces (Table 4). These are: 1) knife-types, with or without worked tips and sometimes bearing partially thinned backs, 2) bifacial tools with peripheral additional sharpened areas and a “*déjeté*” bec with tip management included in the general shaping, 3) partial convergent tools made by few removals, 4) “classical bifaces” with preconceived shaping of a general bifacial volume with two convergent edges and a tip, 5) bifacial cleavers with a transversal cutting edge created by a large lateral removal or several transversal removals included in the general bifacial shaping of the tool. In addition to these LCTs, the series also includes some massive “*rabot*” scrapers.

**Table 4:** Major types of LCTs at la Noira stratum a. Number and size.

	On slab			On flake	Indet.
	Number	Minimum length in mm	Maximum length in mm		
Biface *s.s.*	5	86	153	1 (149-91-35 mm)	2
Backed biface (worked tip)	3	108	153		
Backed bifacial tool	6	105	235	1 (118-82-44 mm)	
Convergent bifacial tool	2	66	120		
Distinct bifacial areas+worked tip	3	88	122		2
Double or multiple bifacial tools	3	90	170		4
Partial bifacial tool	4	57	160		
Bifacial cleaver	3	80	115		
Massive scrapers. Partial LCTs	9	51	115		4

**Figure 10:**
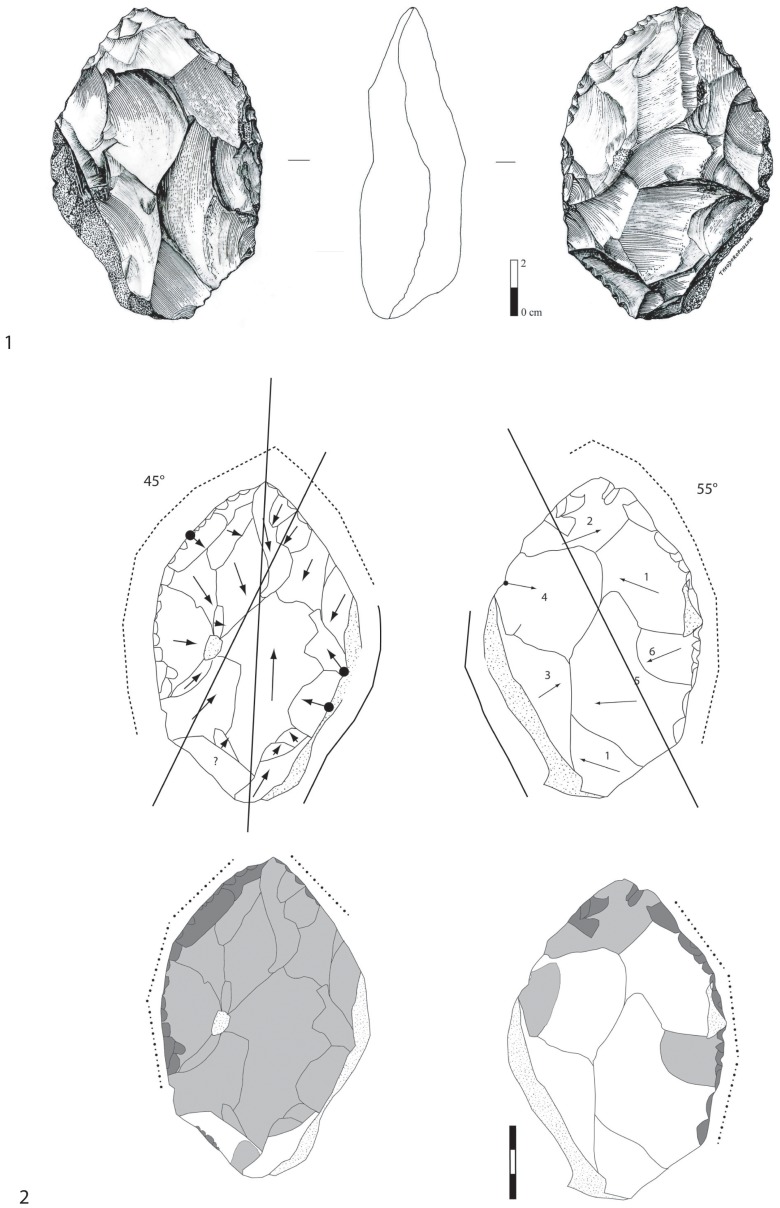
Large Cutting Tool of stratum a. 1.Drawing of a millstone backed LCT (knife-type) on a flake (technical axis different from morphological axis) (drawing A. Theodoropoulou). 2.Interpretative scheme. Series of large removals (grey) completed by final retouch on both cutting edges (dark grey).

1)The backed biface (worked tip) and backed bifacial lateral tool are two variants (Figure 10). The first is a bifacial tool associating two convergent tools managed by bifacial removals and a partial natural lateral back sometimes partially reduced by deep and large removals. They are symmetrical while the section is either symmetrical or plano-convex. There are two successive series of removals, with the second one formatting the upper half of the two convergent edges. The pointed tip is rectified by final retouch. The edge opposite the back is also rectified by continuous retouch suggesting additional functional areas (knife-type) or prehensile parts.

The second variant displays alternate or face by face shaping by a short series of thin removals in order to manage a bifacial partial peripheral cutting edge. Cortical surfaces are largely preserved and tools are not symmetric in shape. The section is symmetrical or plano-convex, except for one twisted piece. The edge opposite the back (unworked or slightly worked) is reworked by a second series of unifacial retouch or short removals. Tool tip management is incorporated into the general shaping. In spite of secondary retouch, the cutting edge remains sinusoidal with angles of approximately 70–80°.

**Figure 11:**
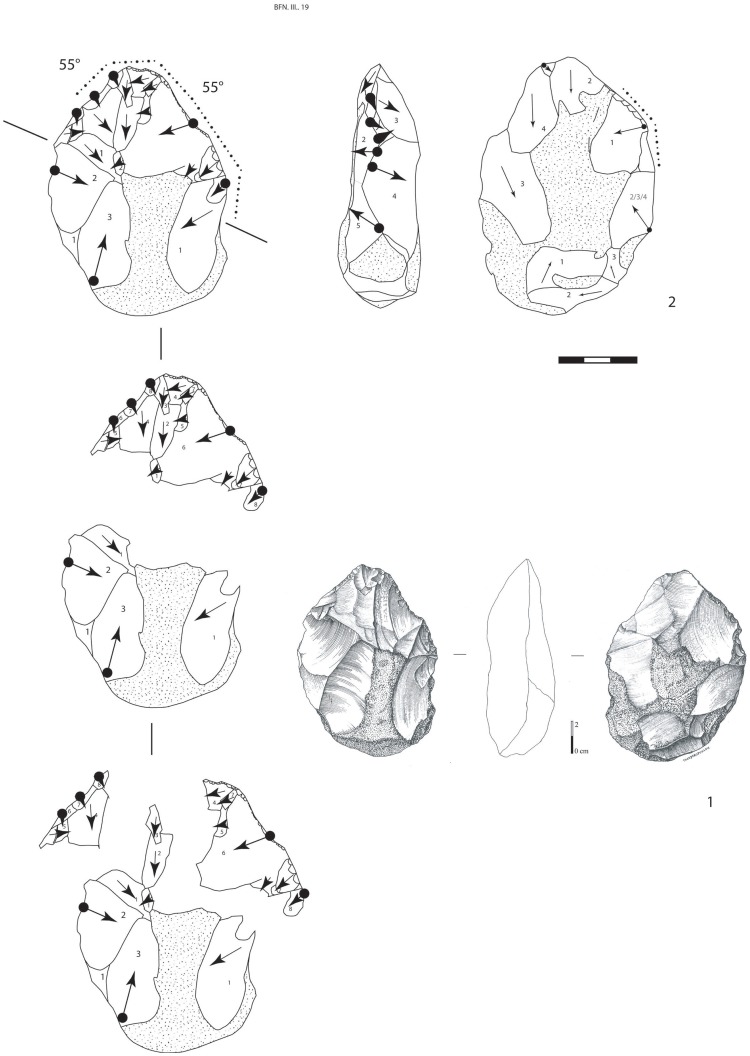
Large Cutting Tool of stratum a. 1.Drawing of a minimally shaped LCT on a millstone nodule with an oval shape (drawing A. Theodoropoulou). 2.Interpretative scheme. View of the different series of removals located on the two convergent edges and the tip.

2)Two groups may be distinguished. Bifacial tools with distinct bifacial areas and a worked tip are characterized by peripheral and invasive bifacial shaping and convergent edges. The tip is carefully worked. Both the organization of the scars and the location and discontinuity of the final retouch suggest distinct sharpened areas. Two notches create a “*déjeté*” bec on the tool tip on two pieces (Figure 11).

**Figure 12:**
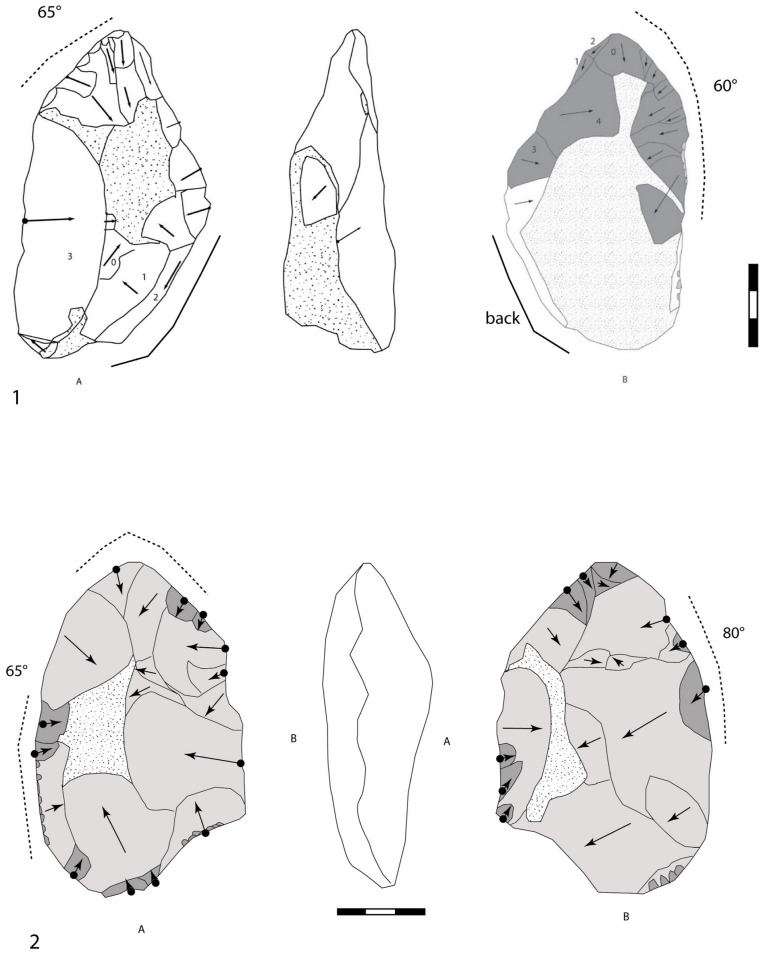
Large Cutting Tools of stratum a. 1. Minimally shaped pointed LCT in millstone. A series of large removals (dark grey) for managing the upper part of the tool. The distal convergent edges and the tip are carefully worked. 2. Extensively shaped pointed LCT in millstone with invasive removals (grey) and edge thinning (dark grey).

Bifacial tools with distinct bifacial areas but without a specific tip are similar to the previous type (Figure 12). We observe: 1) Bifacial peripheral shaping by short and thin removals in one, two or three alternate or face by face series. Tip and base management are included in this general shaping. Micro-retouch is preferentially located on the two convergent edges and the base and three other areas may be distinguished. For one tool, a “nose scraper” is prepared on a lateral edge. Edges are sinusoidal and angle values are varied (from 55 to 85°). Tools are not always symmetric in the same way as the cross-section, 2) Bifacial and invasive shaping with large removals covering the two faces. A second series of short removals are sometimes located on one edge and the tip. Retouch is discontinuous on both edges.

**Figure 13:**
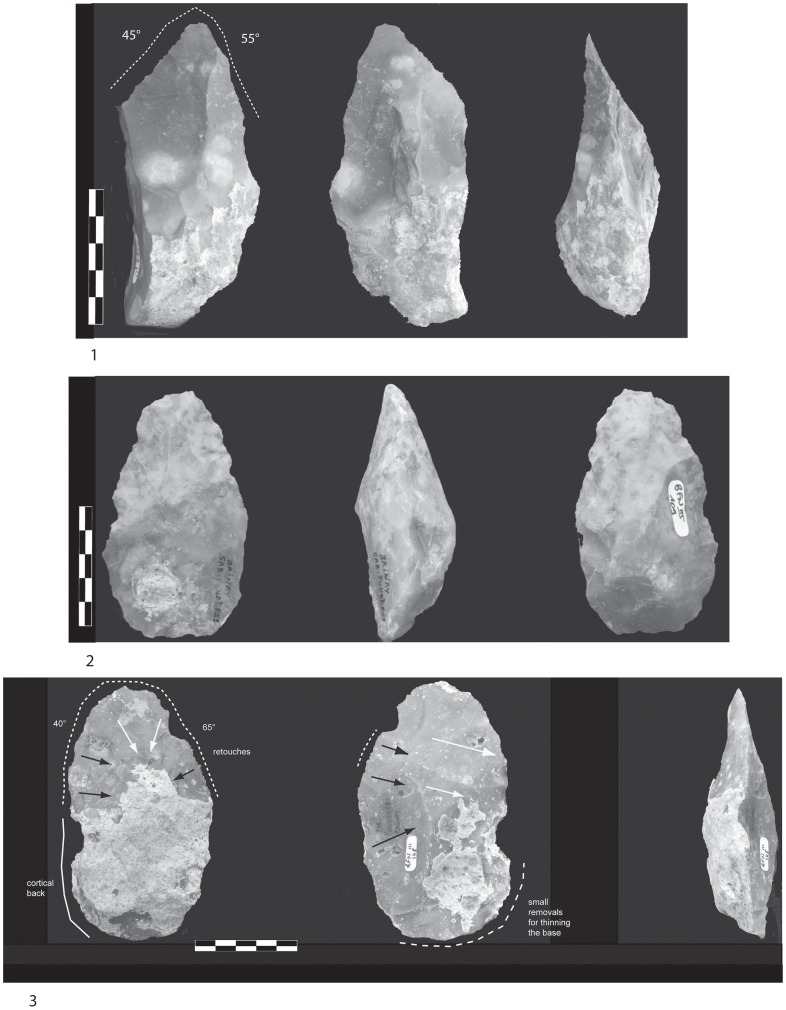
Large Cutting Tools of stratum a. 1. Pointed LCT with shaping of the tip by a series of more or less invasive removals. Diamond shaped cross-section. 2. Extensively shaped LCT with an oval tip. Asymmetrical cross-section. Large removals cover both faces by alternate shaping completed by smaller thinning removals. 3. Minimally shaped symmetrical LCT on a thin millstone slab. Alternate shaping and invasive removals are completed by the thinning of the upper part of the tool. Partial cortical back.

**Figure 14:**
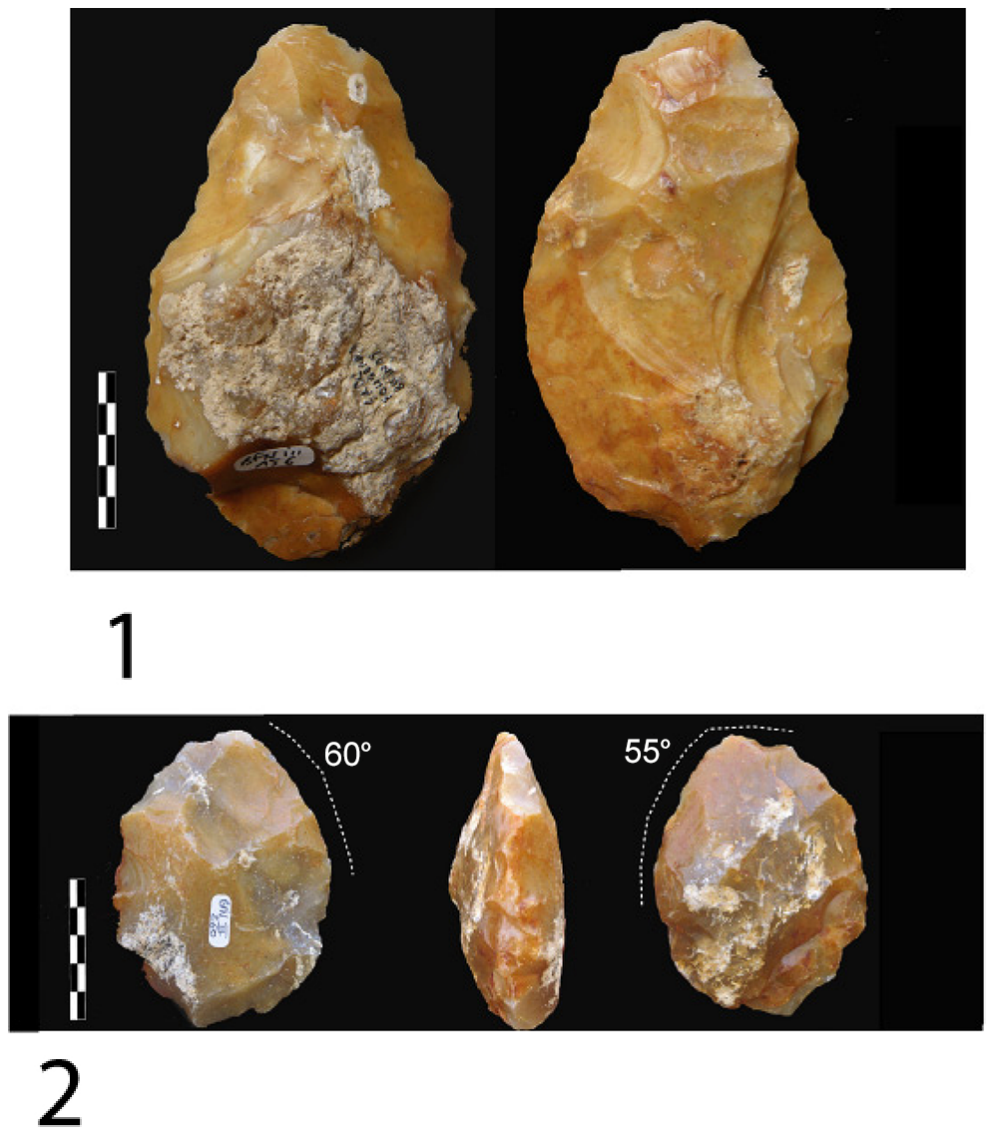
Large Cutting Tools of stratum a. 1. LCT on a millstone flake with large removals on the upper part of the tool and thinning by shorter removals and final retouch of the distal cutting edges and tip. 2. Oval millstone LCT with an asymmetrical cross-section and alternate shaping.

**Figure 15:**
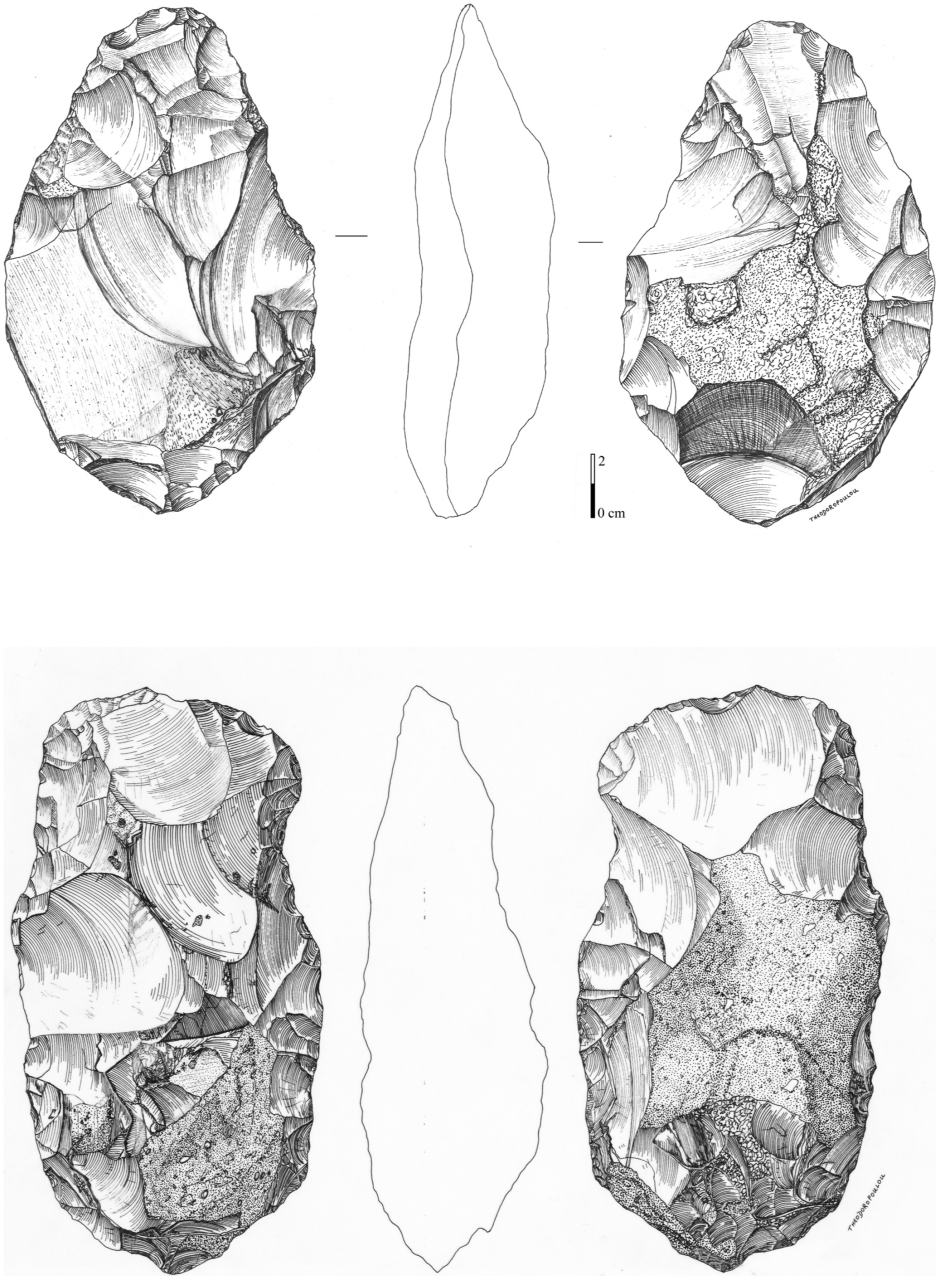
Large Cutting Tools of stratum a. 1.LCT on a millstone flake (drawing of LCT of figure 14). 2.Bifacial cleaver on a millstone slab (drawings A. Theodoropoulou).

3) Partial bifacial tools occupy the upper half or third of a slab (Figure 12, 13). Shaping is made by a small number of short removals except for one tool where a large unifacial removal has been detached in order to shape the tip. The tip morphology is oval and the broken tip of one tool suggests a functional use of the tool extremity. Additional retouch is rare and cutting edge angles vary between 50 and 80°. A second unifacial cutting edge is sometimes prepared on a lateral edge. Unworked single or double backs are preserved.

4) Bifaces *sensus stricto* are symmetrical tools demonstrating the pursuit of a symmetrical bifacial or bilateral equilibrium and a preconceived form (Figures 13, 14, 15). Removals cover both faces, except on flakes where the inferior face is less shaped than the upper surface (Figure 14). Three successive series of scars may be observed, made up of a first series of deep and invasive removals using face by face or alternate shaping mode, and a second series of shorter and thinner removals in order to finish formatting the bifacial volume. Then final retouch is made on parts of the sharp cutting edges and the tip which is worked by complementary short removals. Sections are plano-convex, symmetrical or twisted.

5) The bifacial cleavers are completely covered by numerous invasive bifacial removals on both faces (Figure 15). The transversal cutting edge is created by a large lateral predetermined removal from the initial shaping phase or several transversal removals included in the general bifacial shaping of the tool. The proximal part is oval or rectangular and worked by a series of removals. A second series of small removals rectifies some parts of the two lateral cutting edges and the transversal cutting edge (angle values are systematically close to 50°).

6) Finally, slab fragments (60 to 100 mm long and 11–40 mm thick) are made up of: 1) massive scrapers (“*rabot*” with abrupt or bifacial retouch including scalar retouch on one face). Opposite and inverse retouch on some massive scrapers may be interpreted either as the manufacture of a second tool or a prehensile part of the scraper, 2) partial or total oval bifacial tools, 3) bilateral unifacial tools, and 4) tools such as a “*bec*” made by abrupt retouch.

**Figure 16:**
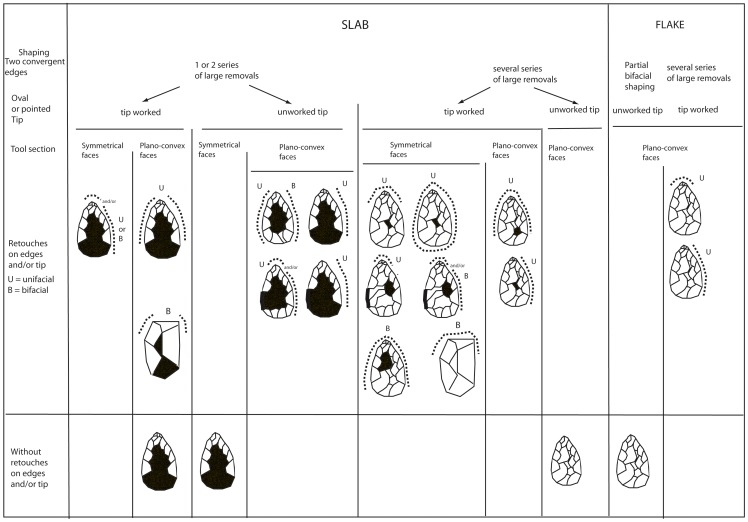
Variability of Large Cutting Tools on slabs and flakes according to shape, extension of removals and location of final retouch on cutting edges at stratum a at la Noira.

One to three series of removals may be identified and these rarely cover both faces completely. The first series is made up of deep hard hammer removals and the second is composed of thin and invasive removals in order to finish the shaping (Figure 16). The shape of the second series of removals indicates the use of a soft hammer. The mode of shaping (alternate or face by face) is partially related to the slab cross-section for the first phases. When the blank is a flake, the inferior face is less covered by scars than the upper face and the tool is minimally shaped. Lastly, on most of the tools, unifacial retouch rectifies some areas of the edges and tip and does not systematically produce regular cutting edges (44% of the edges are sinusoidal). This ultimate retouch could represent resharpening in some cases. It attests to the control of tool edge management. Tips are often thinned by additional short removals. Butts are mainly worked by general shaping removals (38%) or else remain cortical (with a natural slab edge).

From a typological point of view, the LCT morphologies are diverse: cordiform, triangular, oval or amygdaloid-type regardless of the tool category. The tip is either oval or pointed, except for the three bifacial cleavers, whatever the shaping mode. The tip section is triangular, trapezoidal or oval. Neither size nor the elongation index (1–1.5) distinguishes the categories. The thickest part of the LCT is either the butt or the central part of the tool, depending on the aim and intensity of shaping (remains of large cortical surfaces of the slab).

Specifically, 60% of the LCTs are asymmetrical in shape except for bifaces *s.s.* with a bifacial or bilateral equilibrium and a preconceived form. Cross sections are plano-convex or symmetric in relation to the shaping mode, whatever the categories and the final morphological results. They attest to the limited role of slab morphology even if the thinnest slabs were selected for LCTs. Some are twisted. Cutting edge angles generally vary from 50 to 80°.

## Discussion

In spite of the polymorphism of lithic series, whereby each site appears to present specific features, perhaps due to the limited number of available sites in Europe, the la Noira assemblage conforms well with the 600–500 ka European sites with LCTs and the Acheulean (Table 5).

**Table 5:** 800-600 ka European sites [89]–[97].

European sites without LCTs between 800 and 600 ka					
Country	Sites	Ages	MIS	Methods	References
England	Happisburg 3	814–870 ka	21	Biostratigraphy	[4]
		970-936 ka	25	Paleomagnetism	
France	Pradayrol	900 ka	21, 22	Biostratigraphy	[65]
Spain	Gran Dolina TD6	731±63 ka	19,25	Biostratigraphy - TL - IRSL	[89], [91], [94]
		857-780 ka	21	Paléolagnetism - ESR/U-Th	
Spain	Vallparadis	790±230		Biochronology	[47], [92], [93]
		830±130 ka		ESR-ESR/US - Paleomagnetism	
England	Pakefield	700 ka		Paleomagnetism-Amino-acid-Biostratigraphy	[3]
France	Soleilhac	700-500 ka		Biostratigraphy – Paleomagnetism - Tephras	[46], [90], [97]
Italy	Isernia-la-Pineta	610 ka		ESR/US	[46]
		602±2 ka		Ar/Ar	

Sites with *Homo antecessor* remains					
Spain	Gran Dolina TD6	731±63 ka	19,25	Biostratigraphy-TL - IRSL	[89], [91], [94]
		857-780 ka	21	Paleomagnetism - ESR/U-Th	

European sites with LCTs between 700 and 500 ka					
Italy	Nortarchirico	700-600 ka		Tephras - Racemisation	[95]
		640±70 ka		TL	
France	Brinay, la Noira	665±55 ka	16	ESR	[6], [36]
France	Morandière	610±60 ka	15	ESR	[6], [35], [37]
France	Villefranche/Cher	-	15	Stratigraphy	[6], [35], [37]
France	Tautavel-Arago	550 ka		Biostratigraphy – ESR/US - U series	[30]
England	Boxgrove	550 ka	13	Biostratigraphy	[96]
England	Happisburg 1	-	13	Amino-acid - Biostratigraphy	[4]

Sites with *Homo heidelbergensis* remains					
Germany	Mauer	609 ka		Biostratigraphy – ESR/US – IR/RF	[30]
France	Tautavel-Arago	550 ka		Biostratigraphy – ESR/US - U series	[20]

1) As at la Noira, the ratio of LCTs is limited in Europe, and generally varies from 1 to 5%, except for some sites, such as Boxgrove [25], while it is as high as 15% in the Levant (except at the early site of Ubeidiya with rare LCTs) and East Africa.

2) LCT diversity at la Noira is similar to that observed at Arago in levels P-Q, in terms of morphology, diversity of tool types and shaping modes [20]. Some of the LCTs show tip management and a bifacial equilibrium between the two faces. However, flake-cleavers, extensively shaped tools and raw material diversity oppose Arago to la Noira, where no evidence of temporal and spatial fragmentation of the LCT reduction sequence was observed. Although large flakes could have been produced on slabs at la Noira, few were used for shaping and they remained unretouched on the site. LCT diversity and standardization are lower at Notarchirico (levels B, D, F), with both numerous convergent chopping-tools and some rare bifaces *s.s.* [48]. At Boxgrove, ovate tools are standardized with final shaping and possible resharpening using the “*coup de tranchet*” [25].

The same diversity of core technology on local stones is observed on 800–500 ka series without LCTs (Pakefield-700 ka and Happisburg III-900 ka in Great-Britain, Vallparadis and TD6 Gran Dolina-800 ka in Spain, Isernia-504/610 ka in Italy) and with LCTs (Notarchirico-640 ka in Italy, Arago levels P and Q-550 ka, Boxgrove-550 ka in Great-Britain) [46], [3], [4], [20], [47]. La Noira cores are mainly discoidal and they attest to the minor role of raw material shape, like for most of the LCTs of this site. Slabs were selected in accordance with shaping and flaking activities, with the thinnest slabs being reserved for LCTs.

Multiple examples in Europe and in Africa indicate that parameters such as activities [49], [48], [18], occupation duration, raw materials [50], [51], [52], [53], [54], [55], [64], environments or regional variants [38], [56], [57] affect the composition of assemblages, erase inter-site differences and may explain flexibility and some aspects of the diversity of the heavy-duty components (Clactonian *versus* Acheulean) and selective raw material provisioning. Stratum a at La Noira records a workshop context and the use of slabs explains common and specific features observed on the series, both on cores and LCTs. Itconcurs well with other northwestern European sites characterized primarily by the use of flint and the presence of bifacial cleavers, whereas southern European series indicate the use of complementary raw materials and the presence of cleavers on coarse-grained stone flakes.

Since the first identification of the Acheulean by Gabriel de Mortillet (1872) and the earliest use of the term biface by Vayson de Pradennes (1920) in the Somme Valley in France, many definitions and classifications have been proposed, leading to contradictory debates [58], [38]. Some of these discussions are typological and focus on the biface and its morphology, whereby the biface is considered as a “*fossile directeur*”. Others are linked to the handaxe ratio (for instance 40% for Kleindienst [59] or technological aspects such as the ability to produce large flakes which, associated with blocks or pebbles, are then used as blanks for a wide diversity of LCTs, unifacial and bifacial tools, which are either minimally shaped with distinct functional areas or extensively shaped in order to manage a bifacial volume and a tip. There was an application of preconceived morphological templates, which are flexible and adapted to locally available raw materials. This feature may or may not be associated with complex core technology. Since the dichotomy proposed by Leakey [57] between the Developed Oldowan and the Early Acheulean, a large diversity of assemblages and “savoir-faire” have been brought to light both in Africa and Eurasia and the authors of these lithics are commonly referred to as “Acheuleans” [39], [8], [60], [56], [61], [62], [41], [63], [64].

The Early Acheulean is not just defined by the manufacture of LCTs requiring specific and common skills (for instance, the ability to create bilateral equilibrium, maintenance of cross-section and plan-view symmetry and final shaping of the tip and cutting edge) or by the systematic production of large flakes (>10 cm long) using various methods. Many assemblages, where LCTs exist, also yield unsymmetrical tools and lack bifacial tools and minimally shaped LCTs on flakes or blocks. Other parameters are also required to designate the emergence of the Acheulean and new behavioral criteria occasionally materialize as early as 1.5 Ma [41], [44]: higher mobility, more selective raw material gathering when necessary, site location, variety of ecological settings, mobile LCTs and curated pebble tools (multifunctional LCTs, e.g.[44].

Given the shaping technology of the la Noira assemblage, this is an Acheulean assemblage which can be considered as introduced. A local Mode 1 origin and the presence of *Homo antecessor* populations do not appear to be relevant here. It is important to underline the lack of evolutionary features in Mode 1 in Europe before the Acheulean. Very few European sites have yielded remains of pre-bifacial technology with large flakes and partial LCTs. Bogatyri (1.1 Ma) in Russia, Pradayrol (900 ka) and Soleilhac (700-500 ka) have recorded some bifacial endeavors, as observed in East Africa in Oldowan or Developed Oldowan assemblages [65], [66]. The la Noira series does not exhibit Early African Acheulean features (Olduvai, Peninj and Gadeb) such as unifacial tools, picks and numerous heavy duty tools, such as bolas or spheroids [41], [44], [39]. The Acheulean technology at la Noira is advanced and very different from that of the African basal Acheulean. Shaping at la Noira differs from these African assemblages in that it aims to produce many tools with a general LCT bifacial shape by successive series of removals, even if there are also minimally shaped LCTs in the series. Moreover, secondary retouch for rectifying cutting edges and soft hammer use are attested here, whereas in Africa these are common from 1 Ma ago, leading to tool standardization at 700 ka [67], [68], [69]. Thus we cannot postulate that the emergence of new technical behavior at la Noira is issued from a local European substratum, as described in the Early African Acheulean.

Since the la Noira stratum a lithic assemblage shares some features with the 800-500 ka Levantine and African series, two scenarios for the arrival of bifacial technology at 700 ka in Europe are consequently relevant: 1) The arrival of new hominins from the Levant along the Mediterranean coast (*Homo heidelbergensis*?) or new traditions which remained long enough in this part of the world to acquire local features, either through a process of accumulation of behaviors by multiple arrivals or transit between Africa and the Levant inducing behavioral influences. 2) Direct arrivals from Africa through the Levant or North Africa. Direct dispersals from Asia can be excluded due to the specificity of the Indian series and the lack of LCTs in Central Asia and Central Europe. In the absence of clearly dated ancient sites in Spain [19] and even though periods of low sea level are confirmed at ≈700-650 ka [70], little evidence suggests transit from Africa to Gibraltar. The only basis for this is the presence of large flake-cleavers on both sides of the Mediterranean [52], [71]. Flake-cleavers in Spain and southwestern France (including the site of Arago, levels P and Q) are considered as African features and constitute possible evidence of crossing through Gibraltar. In the North of Europe, cleavers are only made by bifacial removals on nodules and we are unable to discern whether they correspond to the same type of tool with a transversal cutting edge managed by a methodology adapted to other raw material shapes, a behavioral specificity of these areas or another kind of tool.

Between 1 Ma and 600 ka ago in Africa, diverse hominins, *Homo erectus* and *Homo rhodensiensis*, were capable of manufacturing bifacial tools [26] during a period considered to represent a technological gap (“classical” Acheulean, soft hammer at 1 Ma ago, occasional blade technology at 500 ka [72]). Assemblages including LCTs display inter-site diversity and tool standardization (e.g. Thomas Quarry I level L −1 Ma, Olorgesailie-900-600 ka, Garba XII level J-900 ka, Gombore II-800 ka, Tighenif-800 ka [56], [73], [74], [75], [76]. Twisted LCTs from la Noira resemble obsidian bifaces from Gombore [42] and shaping modes are similar to those employed at Isenya-700 ka [69]. From the Levantine corridor to the Arabian Peninsula, the dearth of sites prevents us from reconstructing Acheulean filiations. Even so, sites such as GBY-790 ka, El Meirah and Latamne-700-500 ka in Syria, Karakhach I in Armenia, Joubb Jannine (900 ky) in Lebanon or El Kowm Basin localities suggest a mosaic of regional variants and layers of local traditions and traditions with African affinities [49], [9], [77], [78], [79], [80].

## Conclusion

The lithic assemblage of la Noira provides evidence of the Acheulean tradition at 700 ka in the center of France. The age of 700 ka establishes an early Acheulean presence in Europe and modifies our current vision of the initial peopling of northern Europe. The Acheulean is thus older than previously assumed and the oldest evidence is no longer located in southern Europe.

The hominins who made the LCTs found in stratum a were present at la Noira after the period of river incision, at the beginning of a glacial climatic period (MIS 16), before the pleniglacial phase, as demonstrated by the cryoturbation of the archaeological level. They settled at the bottom of the slope along the river and beside suitable local slabs. The archaeological remains were then overlaid by fluvial sand attributed to the next interglacial stage. Seven ESR ages obtained on these deposits point towards the attribution of the sequence to the MIS 16/15 climatic cycle.

The la Noira stratum a yielded wide LCT diversity and a structured core technology as early as 700 ka. The lithic assemblage suggests pioneering phases of bifacial technology before 500 ka in Europe. Low hominin densities, adaptation to Europe, distance from the core area and perhaps hominin diversity, like in East Africa, could account for this multiplicity [81], [82]. LCT diversity, relative independence from the natural morphology of the raw material and different degrees of planning in the knapping process provide evidence of an external Acheulean tradition at la Noira, even though there is no temporal or spatial fragmentation of the LCT reduction sequence and the procurement system is geared almost exclusively towards local stone (workshop context).

These pioneering phases would have taken place on a Mode 1-type substratum, possibly encountering Mode 1 groups even if population densities were low, and adapting to different vegetal and animal landscapes than those in the Levant or Africa. Environmental and chronological data from northwestern sites suggest occupations during climatically favorable periods (temperate climate). The specific time period in question here is centered on the Middle Pleistocene Transition (MPT), after the Brunhes-Matuyama shift at 800 ka leading to an extension of the grassland habitat into higher latitudes, thereby opening or closing corridors [83], [84], [85], [86], [87]. Cyclical climate changes (glacial/interglacial) would have led to successive depopulation or extinction, and the subsequent recolonization before and between the MIS 16 and 12 cold events [27]. The second later transition (Mid-Brunhes Event –MBE), occurring between MIS 13 and 11, would have allowed for a more widespread diffusion of elaborate bifacial manufacture techniques. Vital information is missing for determining pathways and timing, and filiation appears to be impossible to resolve due to the influence of activities and environmental patterns on behavior. Relationships with faunal turnover across Eurasia are not always evident, due to a lack of clear mammalian dispersals from Africa to Eurasia and through the Saharan belts. Relationships between changes in carnivore associations (around 500 ka) and the demise of feline megahunters as early as 1 Ma ago, climatic changes and hominid dispersals advance contradictory arguments [88]. These environmentally linked changes could have promoted sporadic hominin expansion to Europe aided by new technics and new social organization [61], [79]. It is not yet clear precisely which advantages *Homo heidelbergensis* held over *Homo antecessor*, although a larger brain; “brain expansion scenario”, and a new technology may hold part of the answer.
